# Rheological Behavior of Tomato Fiber Suspensions Produced by High Shear and High Pressure Homogenization and Their Application in Tomato Products

**DOI:** 10.1155/2018/5081938

**Published:** 2018-03-18

**Authors:** Yong Wang, Ping Sun, He Li, Benu P. Adhikari, Dong Li

**Affiliations:** ^1^Beijing Advanced Innovation Center for Food Nutrition and Human Health, Beijing Technology & Business University, 11 Fuchenglu, Beijing 100048, China; ^2^Beijing Key Laboratory of Nutrition & Health and Food Safety, COFCO Nutrition & Health Research Institute, No. 4 Road, Future Science & Technology Park, Beijing 102209, China; ^3^Processing Technology Research Center for Tomato, COFCO Tunhe, Changji, Xinjiang 831100, China; ^4^School of Applied Sciences, RMIT University, City Campus, Melbourne, VIC 3001, Australia; ^5^College of Engineering, China Agricultural University, P.O. Box 50, 17 Qinghua Donglu, Beijing 100083, China

## Abstract

This study investigated the effects of high shear and high pressure homogenization on the rheological properties (steady shear viscosity, storage and loss modulus, and deformation) and homogeneity in tomato fiber suspensions. The tomato fiber suspensions at different concentrations (0.1%–1%, w/w) were subjected to high shear and high pressure homogenization and the morphology (distribution of fiber particles), rheological properties, and color parameters of the homogenized suspensions were measured. The homogenized suspensions were significantly more uniform compared to unhomogenized suspension. The homogenized suspensions were found to better resist the deformation caused by external stress (creep behavior). The apparent viscosity and storage and loss modulus of homogenized tomato fiber suspension are comparable with those of commercial tomato ketchup even at the fiber concentration as low as 0.5% (w/w), implying the possibility of using tomato fiber as thickener. The model tomato sauce produced using tomato fiber showed desirable consistency and color. These results indicate that the application of tomato fiber in tomato-based food products would be desirable and beneficial.

## 1. Introduction

Tomato (*Lycopersicon esculentum* Mill.) is one of the most popular fruits over the world because of its unique visual appeal, taste, and nutritional value as it contains ascorbic acid (vitamin C) and lycopene [1]. Processed tomato products such as purees and sauces are a primary source of tomatoes in contemporary diet. Considerable research has been undertaken in the past to quantify and elucidate the natural consistency and structure of tomato products [2].

From a structural point of view, most tomato products are aqueous dispersion containing aggregated or disintegrated cells and cell wall material dispersed in water soluble tomato components. The consistency of processed tomato products arises from the cell wall components such as cellulose, semicellulose, pectin, and interactions among these components [2]. Cellulose is major component of vegetable cell wall suspensions and it is also the main component that affects the rheology of processed tomato products. Pectins are embedded naturally within the cellulose backbone and they are also found in the serum phase. They are known to contribute to the structure of tomato products significantly depending on the processing conditions [3–6].

Homogenization is a key processing step in the production of ketchup, sauces, and other tomato products. The homogenization process decreases the mean particle size of the tomato suspensions and imparts smoother texture and higher viscosity. It also alters the nature of the suspensions network and increases the viscosity of the suspensions [7, 8]. During homogenization, tomato pulp is subjected to very high turbulence, shear, cavitation, and impact when it is forced through the homogenizer [9]. The homogenization process was found to alter particle size distribution, pulp sedimentation behavior, serum cloudiness, color, and microstructure of tomato juice, by disrupting the suspended pulp particles [10]. High pressure homogenization was reported to decrease the particle size due to the disruption of matrix and increase tomato product's Bostwick consistency, probably due to the formation of fiber network [11]. The large discrete cells and cell fragments of tomato suspensions were easily degraded by homogenization which resulted into higher water-holding capacity [6, 7, 11]. The high pressure homogenization reduced the mean particle size and narrowed the particle size distribution thereby increasing the total surface area and the interaction among the particles [12]. Bengtsson et al. reported that the nonhomogenized tomato suspensions had swollen cell structure with relatively few cell aggregates; however, the homogenized suspensions contained large number of degraded cell fragments [13].

Tomato peel is a by-product of tomato industry and fiber is extracted from tomato peel using chemical method [14]. Tomato peel fiber contains about 80% of total dietary fiber (mainly water insoluble fiber) much higher than other vegetable by-products [15]. Due to its unique chemical composition and functional properties, tomato peel fiber can be used as a food supplement to improve physical, chemical, and nutritional properties of food products. However, the color and flavor of tomato peel fiber must be considered carefully to avoid their negative impact on the sensorial characteristics of the final products [16]. To date the tomato fiber has received very little research attention despite its ability to contribute to desirable food texture and good mouth feel.

To the best of our knowledge, there is no study on the effect of high shear and high pressure homogenization on the tomato fiber. Thus, this study aimed to study the effects of high shear and high pressure homogenization on the morphological and rheological properties of tomato fiber suspensions. We also compared the morphological, rheological, and color parameters of homogenized tomato fiber suspensions with those of commercial tomato ketchup and a model tomato sauce formulated for comparison. We believe that the findings presented in this paper will provide better understanding of the functional properties of tomato fiber and help broaden its application as an important thickening ingredient in food industry.

## 2. Materials and Methods

### 2.1. Materials

The tomato fiber sample was kindly provided by COFCO Tunhe Co. Ltd., Beijing, China. The solid content of this fiber sample was determined and found to be 4.80% (w/w). This fiber sample contained 2.11% (w/w) insoluble dietary fiber as tested following the AOAC Official Method 991.43 [17] and 1.12% (w/w) protein as tested using China's national food safety standards [18]. The tomato fiber was produced by concentrating and separating the solid part out of the tomato paste (without tomato peels or seeds), by using high speech rotary mechanical instrument.

The food grade tomato paste (29.0° Brix cold break), tomato ketchup, sugar, soybean fiber, and salt used in this study were provided by COFCO Tunhe Co. Ltd., Beijing, China. Deionized water was used to prepare samples.

### 2.2. Mechanical Treatments

The tomato fiber suspensions were prepared in four concentrations (0.1%, 0.25%, 0.5%, and 1%, w/w) by mixing raw tomato fiber with adequate amount of deionized water as calculated based on the moisture content of tomato fiber.

The shearing treatments were carried out using a laboratory disperser (IKA Ultra-Turrax T25, Germany). The tomato fiber suspensions were subjected to 3400 rpm, 5000 rpm, 8000 rpm, 10000 rpm, 12000 rpm, and 14000 rpm for 12 minutes each.

The above-mentioned sheared samples were homogenized using a high pressure homogenizer (ATS AH100D, Shanghai, China), which is a lab-scale homogenizer equipped with valve. The maximum pressure of this homogenizer is 140 MPa. The homogenization was carried out for 2 passes at 0 MPa, 5 passes at 5 MPa, and then another 5 passes at 10 MPa.

### 2.3. Determination of Morphology

Twenty milliliter of untreated, sheared, and homogenized suspensions were separately placed in colorimetric tubes. Images were captured with a digital camera in order to compare the appearance of these suspensions. The microscope images of all the above-mentioned samples were acquired. Very small drop of each sample was placed on a microscope slide and the pictures were taken using a microscope (Olympus CX31, Japan) at 100x and 400x magnification.

### 2.4. Rheological Measurements

Rheological measurements were performed using AR2000ex rheometer (TA Instruments Ltd., Crawley, UK). This is a controlled stress, direct strain, and controlled rate rheometer coming with torque range from 0.0001 to 200 mN·m and high stability normal force from 0.01 to 50 N. The parallel plate was used for all the tests. The temperature was controlled by a water bath connected to the Peltier system in the bottom plate. A thin layer of silicone oil was applied on the edges of samples in order to prevent evaporation. The linear viscoelastic region was determined for each sample through strain sweeps at 1 Hz (data not shown). Viscoelastic properties [storage (*G*′), loss (*G*′′) modulus, and loss tangent (*δ*)] of samples were determined within the linear viscoelastic region. The samples were allowed to equilibrate for 2 min before each measurement.

The steady shear tests were performed at 25°C over the shear rate range of 0.01–100 s^−1^ to measure the apparent viscosity. A steel cone geometry (60 mm diameter, 59* μ*m gap) was chosen for these measurements, since cone geometry is more preferable for viscosity measurement.

The frequency sweep tests were performed at 25°C over the angular frequency range of 0.1–10 rad/s. The strain amplitude of these frequency sweep measurements was selected to be 1% according to the strain sweep results (data not shown) in order to confine these tests within linear viscoelastic region. An aluminum parallel plate geometry (40 mm diameter, 1 mm gap) was chosen for these measurements.

Creep experiments were carried out at a fixed shear stress of 7.958 mPa at 25°C. The variation in shear strain in response to the applied stress was measured over a period of 2 min. An aluminum parallel plate geometry (40 mm diameter, 1 mm gap) was chosen for these creep measurements.

### 2.5. Preparation of Tomato Sauce

The formulation of tomato sauce samples used in the first round of tests is provided in Table 1. The tomato paste and homogenized tomato fiber or soybean fiber were mixed according to this formulation. Required amount of water was added to make the mass of the sample to be 110 g. The homogenized tomato fiber with 2.5% concentration was prepared as described in Section 2.2.

The formulation of tomato sauce for second round of tests is shown in Table 2. Two hundred grams of sauce was prepared for each formulation by measuring and mixing ingredients listed in Table 2. The mixture was then heated at 95°C for 10 min in a water bath with continuous stirring. The sauce container was covered during heating to minimize the evaporation of water. The sauce was finally cooled down to ambient temperature.

### 2.6. Analysis of Physicochemical Properties

Bostwick consistency was determined using a standard 24 cm Bostwick Consistometer with 48 × 0.5 cm graduations (Endecotts ZXCON-CON1, London, UK). Seventy-five mL of sample was used to perform these tests. As the fluid flows down the instrument, the measurements were carried out after 30 seconds.

Colorimetric tests were performed using a spectrophotometer (Hunter Lab UltraScan VIS, Reston, US) in transmission mode. The samples were filled into a 10 mL quartz transmission cell with 10 mm path length. The *L*, *a*, and *b* values were calculated by the averaging the data of triplicate runs. The suspensions were shaken to achieve uniformity in color immediately before measurement.

The pH and total acidity of samples were measured using an automatic acid analyzer (Metrohm 877 Titrino plus, Switzerland).

In order to measure the Bostwick consistency, color, pH, and total acidity of the tomato source samples, the total soluble solids content was adjusted to 12.5° Brix in order to keep the same test condition. A refractometer (Atogo RX-5000*α*, Japan) was used for this purpose.

### 2.7. Statistical Analysis

All of the above-mentioned tests were carried out in triplicate. The rheological data was obtained directly from the AR2000ex rheometer software (TA Instruments Ltd., Crawley, UK). The averaged value of triplicate runs was reported as the measured value along with the standard deviation.

## 3. Results and Discussion

### 3.1. Effect of Homogenization on Suspension Morphology

**Figure 1:**
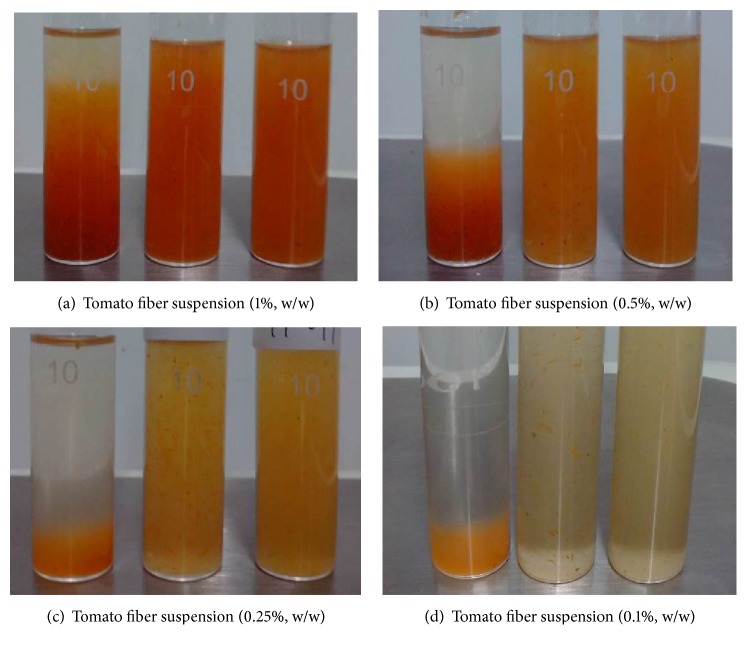
Photographs of tomato fiber suspensions at different concentration. In each photograph from left to right, untreated sample, high shear homogenized sample, and high pressure homogenized sample.

The effect of mechanical treatment on the appearance of tomato fiber suspensions at solid concentrations of 0.1–1.0% (w/w) is shown in Figure 1. The solid content was easily precipitated towards the bottom of the tube in all of the untreated samples irrespective of fiber concentration and the amount of sediment increased with increase in fiber concentration. The uniformity of suspensions greatly increased after shear homogenization or high pressure homogenization. The uniformity was relatively poor in shear homogenized samples at 0.1% and 0.25% (w/w) concentration compared with that of high pressure homogenized samples. The uniformity of suspensions produced by shear homogenization and high pressure homogenization was similar at 0.5% and 1.0% (w/w). It has been previously reported that the more stable network structure can be formed in tomato fiber suspension when homogenized at 9 MPa [7]. It can be observed from photographs presented in Figure 1 that the shear homogenization affects only a part of the tomato fiber, most likely from tomato flesh. The fibers from tomato pericarp could only be fragmented under high pressure homogenization. The structural features of tomato fiber particles are drastically altered by the high pressure homogenization. It has been reported that the homogenized tomato fiber suspensions consisted of smashed cellular material which eventually formed fibrous-like network while the nonhomogenized suspensions consisted of a mixture of whole cells and dispersed cell wall materials [6].

**Figure 2:**
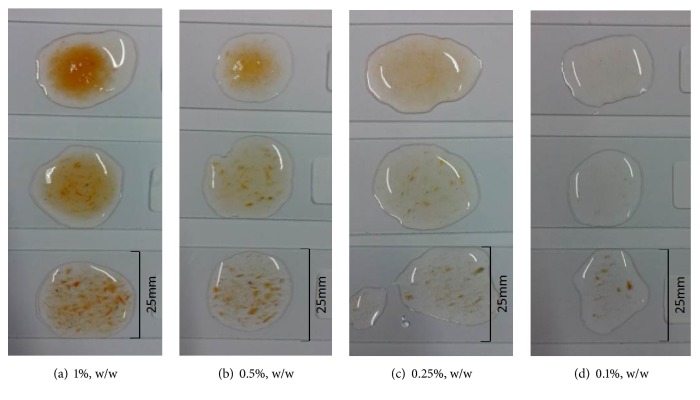
Microscopic photographs of tomato fiber suspensions showing distribution of fiber solids at different concentration. In each photograph from bottom to top, untreated sample, shear homogenized sample, and high pressure homogenized sample.

The distribution of solids in tomato fiber suspensions is illustrated in Figure 2. Dark red discrete particles are observed in untreated and high shear homogenized samples at all concentrations, while the high pressure homogenized sample showed much better uniformity in solid distribution. The high pressure homogenized suspensions containing 0.5% or 1% (w/w) fiber began to exhibit water-holding properties, indicated by the increased height of tomato fiber sample on the glass (picture not shown). It was reported earlier that the homogenized tomato fiber suspensions showed higher water-holding capacity albeit at much higher solid concentrations (10% to 21.7%) [13]. This increased water-holding capacity would be a beneficial whenever the tomato fiber is used as an ingredient to impart desired texture in food products. The information presented in Figures 1 and 2 agree with the findings in an earlier study [19] that the unhomogenized tomato juice showed whole cells with intact membranes and characteristic lycopene crystals while the homogenized samples showed large number of small particles composed of cell walls and internal constituents suspended in the juice serum.

The values of colorimetric parameters (*L*, *a*, and *b*) of unhomogenized tomato fiber suspensions at different concentration are presented in Table 3. The *L* and *b* values decreased with increase in fiber concentration while the *a* value showed substantial increase. The *a*/*b* value, which is of vital importance in the tomato processing industry, significantly (*p* < 0.05) increased with the increase in concentration. The *a*/*b* value of 2% (w/w) tomato fiber suspension suggested that this formulation has desirable color for potential application in tomato sauces. It has also been reported in an earlier study that the values for *L*^*∗*^, *a*^*∗*^, and *b*^*∗*^ increased with the increase in homogenization pressure indicating that the tomato fiber suspensions became more saturated in red and yellow color [10].

**Figure 3:**
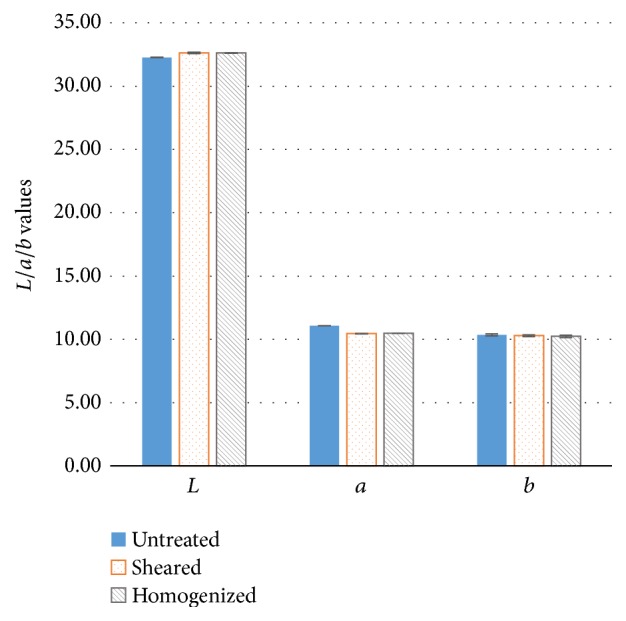
Effect of high shear and high pressure homogenization on the colorimetric parameters (*L*, *a*, and *b*) of 1% (w/w) tomato fiber suspension.

The effects of high shear and high pressure homogenization on the 1% (w/w) tomato fiber suspension are shown in Figure 3. None of the *L*, *a*, or *b* parameters was significantly (*p* > 0.05) affected by the high shear homogenization or high pressure homogenization.

**Figure 4:**
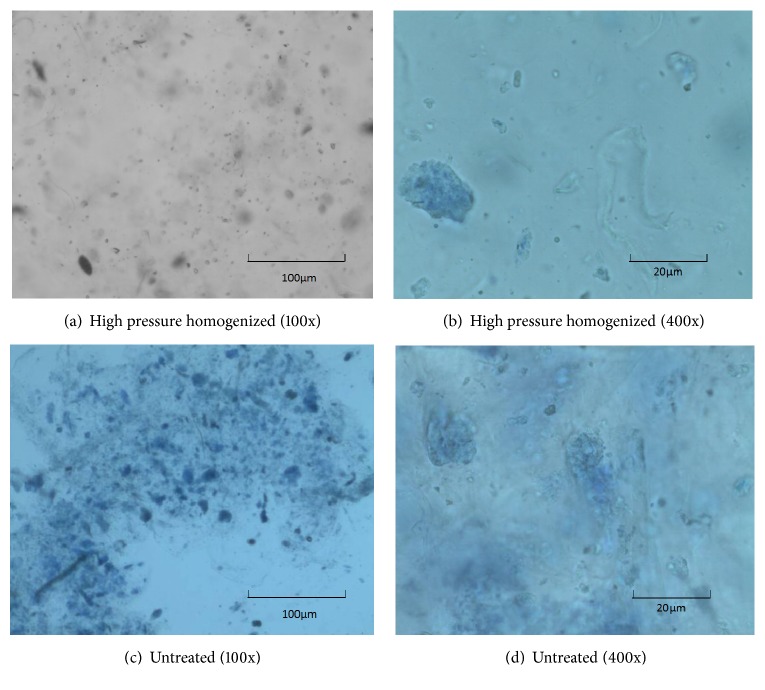
Microscopic photographs of 1% (w/w) tomato fiber suspension before and after mechanical treatment.

In order to illustrate the morphological changes caused by homogenization, the microscopic photographs of 1% (w/w) tomato fiber suspension are shown in Figure 4 before and after homogenization. After high pressure homogenization, the solid tended to be evenly distributed at microscopic level (Figure 4(a)). The tomato fiber suspension showed a fibrous morphology with high degree of uniformity resembling a solution with negligibly very small amount of suspended solid after high pressure homogenization (shown in Figure 4(b)). The control samples showed unperturbed cells with intact membrane and the characteristic lycopene crystals. The homogenized samples showed a large number of small cell wall particles and internal cell constituents suspended in the juice serum which agreed with Kubo et al. observation [10]. It has been reported that no intact cells were observed in tomato pulp subjected to high pressure (479 bar) homogenization and the internal cell constituents were found to be uniformly distributed in the homogenized pulp [9].

### 3.2. Effect of Homogenization on Rheological Properties

**Figure 5:**
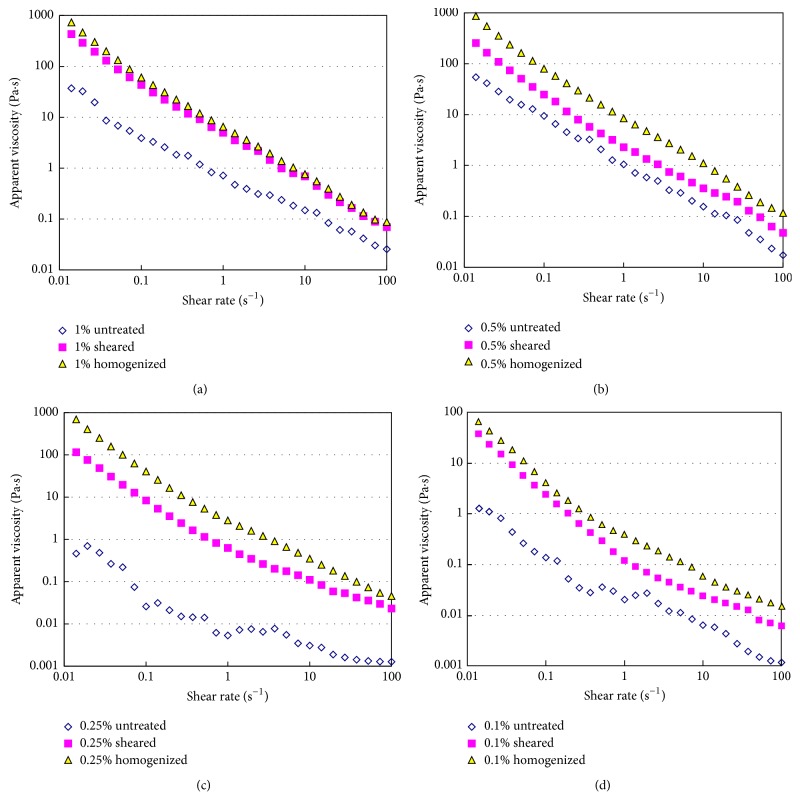
Flow behavior of tomato fiber suspensions before and after high shear or high pressure homogenization. Tomato fiber concentration: (a) 1%; (b) 0.5%; (c) 0.25%; (d) 0.1%; all in (w/w) basis.

As shown in preceding section, the texture of tomato fiber suspensions could be significantly modified by homogenization. The effect of high shear and high pressure homogenization on the apparent viscosity is shown in Figure 5. All the tomato fiber suspensions showed shear-thinning behavior regardless of the concentration before and after homogenization. The apparent viscosity of all the samples increased with the increase in fiber concentration. The high shear homogenization significantly (*p* < 0.05) increased the apparent viscosity compared to the untreated sample. The application of high pressure homogenization increased the apparent viscosity the most (Figures 5(a)–5(d)). Augusto et al. reported that the viscosity of tomato juice (4.5° Brix) increased when the homogenization pressure increased from 50 MPa to 150 MPa [12]. Similar effect of high pressure homogenization which was on tomato suspensions was reported in various studies [6, 7, 20]. The cell wall of tomato cells could be broken even at moderate shear and this rupture is linked with the increase in viscosity.

The power law model (see (1)) was used to predict the variation of apparent viscosity with shear rate of tomato fiber suspensions.

(1) $$\begin{array}{c} \mu_{a}=K{\dot{\gamma }}^{n-1}, \end{array}$$

where *μ*_*a*_ is the apparent viscosity (Pa·s), $\dot{\gamma }$ is the shear rate (s^−1^), *K* is consistency coefficient (Pa·s^n^), and *n* is the flow behavior index (dimensionless). The values of *K* and *n* for all the test samples were determined by fitting (1) to experimental apparent viscosity versus shear rate data presented in Figure 5 and are presented in Table 4. The flow behavior index (*n*) depends on the distribution of small and large particles and the rheology of the suspending fluid, while the consistency coefficient (*K*) depends on the maximum packing fraction (*φ*_*m*_) and the distribution of small and large particles [21]. The *K* value increased very strongly with the increase of fiber concentration in all samples. The *n* value, which is indicator for shear-thinning behavior, was the lowest in pressure homogenized samples, the highest in the untreated samples, and intermediate in high shear homogenized samples at a given concentration. This means that the high pressure homogenized samples are most susceptible to shear thinning.

**Figure 6:**
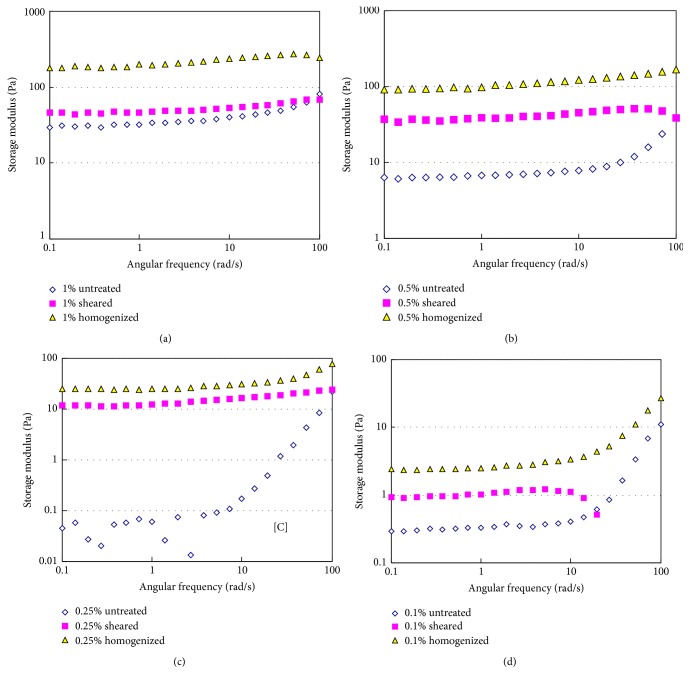
Storage modulus (*G*′) of tomato fiber suspensions before and after homogenization. Tomato fiber concentration: (a) 1%; (b) 0.5%; (c) 0.25%; (d) 0.1% (w/w).

The values of storage modulus (*G*′) of the homogenized and unhomogenized tomato fiber suspensions are shown in Figure 6. Both the homogenized and unhomogenized samples showed a slight increase of *G*′ with the increase in angular frequency. At lower fiber concentrations (0.1%–1%), the *G*′ value of the high shear homogenized suspension increased more strongly compared to the unhomogenized sample. The increase of the *G*′ value was the strongest in high pressure homogenized suspension which is similar to the variation of apparent viscosity with shear rate. This observation agrees with the earlier report that the homogenization process increases both storage and loss modulus of tomato suspension [7, 19].

**Figure 7:**
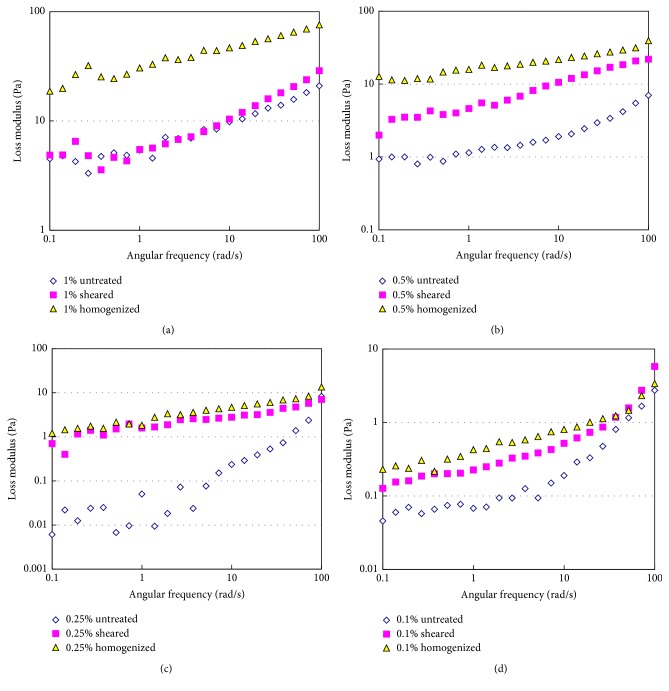
Loss modulus of tomato fiber suspension before and after homogenization. Tomato fiber concentration: (a) 1%; (b) 0.5%; (c) 0.25%; (d) 0.1% (w/w).

The loss modulus (*G*′′) of tomato fiber suspensions are presented in Figure 7. The *G*′′ values increased with the increase in tomato fiber concentration. Both high shear and high pressure homogenization processes significantly (*p* < 0.05) increased the *G*′′ values. The high pressure homogenization appears to be more effective in increasing *G*′′ values as a function of angular frequency. All suspensions exhibited solid-like behavior with *G*′ being higher than *G*′′. Augusto et al. studied the effect of high pressure homogenization (up to 150 MPa) on the viscoelastic properties of tomato juice and found both *G*′ and *G*′′ when the juice was homogenized [22]. The increase in homogenization pressure was also found to increase both *G*′ (75.4 Pa to 212.2 Pa) and *G*′′ (from 49.8 Pa to 80.9 Pa) in tomato suspensions [13].

**Figure 8:**
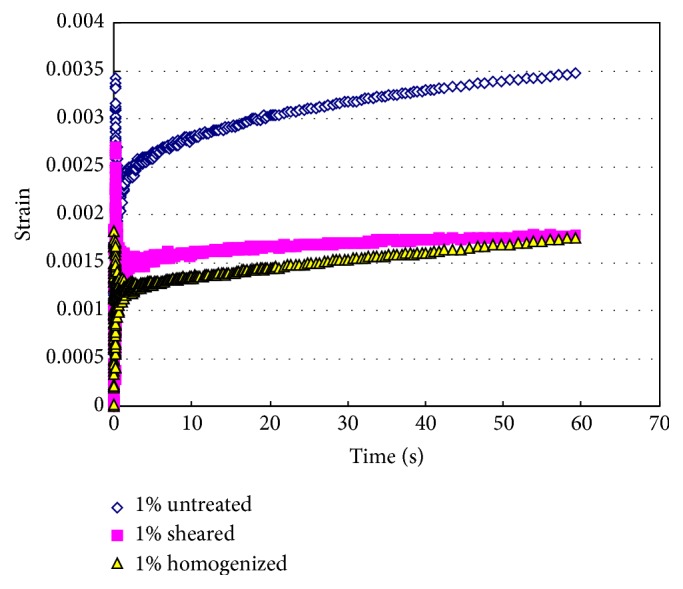
Creep diagrams of tomato fiber suspensions before and after homogenization. Tomato fiber concentration: 1% (w/w).

The effect of homogenization on the creep behavior of tomato fiber suspensions is presented in Figure 8. At 1% (w/w) concentration, homogenized suspensions deformed less that the control sample under the same applied stress. The high pressure homogenized sample had the largest resistance to the applied stress among all the samples. This further indicates that homogenization helps build a stronger texture in the tomato fiber suspension, which could utilized to formulate food products with desirable texture. Figure 8 also shows that the slope of the creep curve is much smaller compared to that of the control sample. This indicates that high shear and high pressure homogenized suspensions achieve an equilibrium state to maintain their solid-like structure sooner compared to the unhomogenized suspension. At the same stress, the unhomogenized suspension would continue to deform. This observation is consistent with earlier publication which reported that the homogenized tomato juice reduced the compliance of tomato juice due to stronger internal structure [19].

Based on all the rheological data presented above, it could be concluded that the rheological properties of tomato fiber could be significantly altered by the application of high shear or high pressure homogenization. The homogenized suspensions had higher apparent viscosity, higher *G*′, and *G*′′ and they could withstand larger external force and could maintain the solid-like structure better.

### 3.3. Comparison with Tomato Ketchup

**Figure 9:**
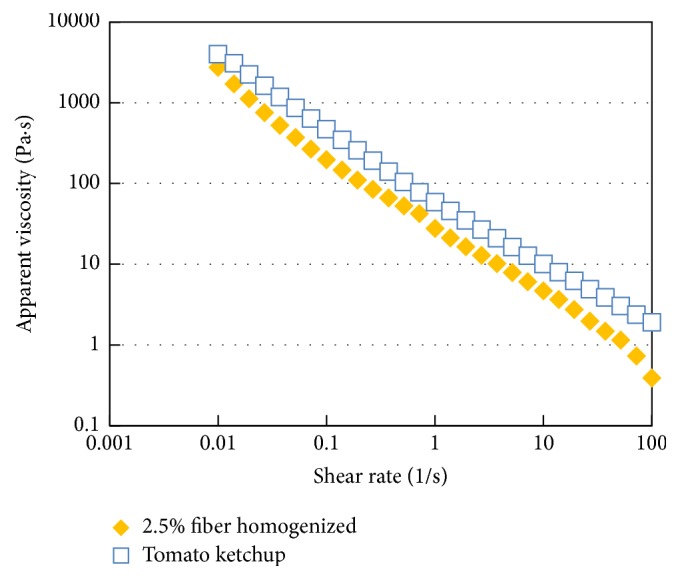
Apparent viscosity versus shear rate profiles of homogenized tomato fiber suspension and tomato ketchup (30° Brix).

Viscosity is a key indicator of quality of tomato paste and ketchup based on which consumers make their purchasing decision [23]. The apparent viscosity of high pressure homogenized tomato fiber suspension at 2.5% (w/w) fiber concentration was compared with that of tomato ketchup of 30° Brix (Figure 9). Despite the large difference in solid concentration between the two samples, they show similar shear-thinning behavior and comparable apparent viscosity. Thus, the tomato fiber can replace other thickeners which might have been used in tomato ketchup, for example, pectin or xanthan gum.

**Figure 10:**
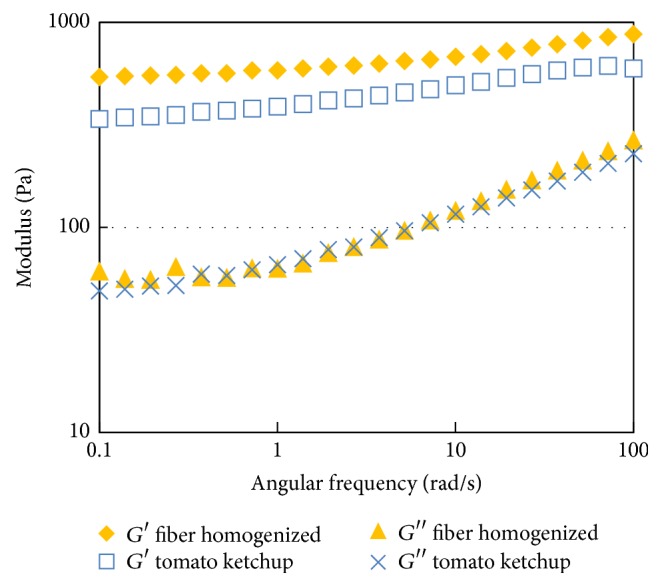
Storage and loss modulus of high pressure homogenized tomato fiber suspension (2.5%, w/w) and tomato ketchup (30° Brix).

The *G*′ and *G*′′ versus angular frequency curves of high pressure homogenized tomato fiber suspension (2.5%, w/w) and tomato ketchup (30° Brix) are presented in Figure 10. The curves of *G*′′ versus angular frequency of these two samples were almost identical. The *G*′ versus angular frequency curves of these samples bear similar trend. The storage modulus of the homogenized fiber suspension was higher than that of the tomato ketchup within the entire angular frequency range. This indicated that the fiber suspension had stronger three-dimensional structure to resist external stress than the tomato ketchup. The viscoelastic characteristics of tomato sauce or ketchup are reported to depend on the diameter of the suspended particles water insoluble solids content [24]. The data presented in Figure 8 indicates that tomato fibers might be better choice if firmer or more solid-like texture is required.

**Figure 11:**
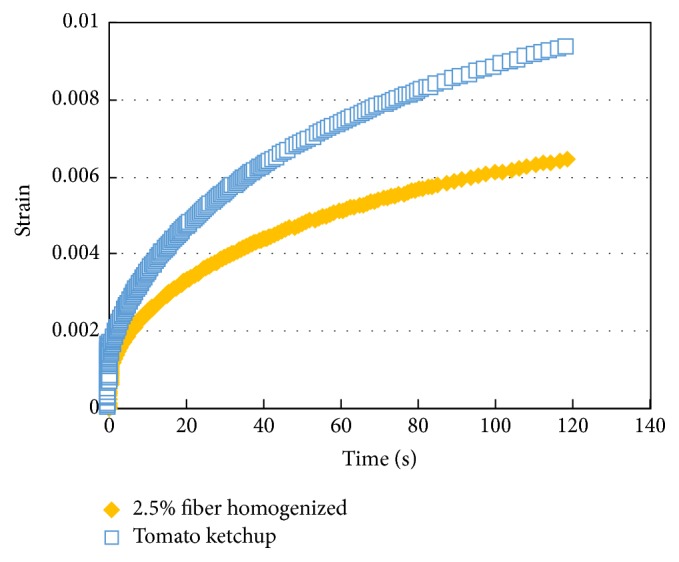
Creep diagram of high pressure homogenized tomato fiber suspension (2.5%, w/w) and tomato ketchup (30° Brix).

The creep diagrams of high pressure homogenized tomato fiber suspension (2.5%, w/w) and tomato ketchup (30° Brix) are shown in Figure 11. The tomato fiber suspension deformed to a lesser extent than the tomato ketchup corroborating the fact that the tomato fibers provide firmer texture than the ketchup, although the texture is also affected by concentration. According to a sensory evaluation data reported in earlier study the tomato suspension homogenized at 90 bar had significantly better thicker and smoother texture and significantly weaker graininess compared with the untreated sample [13].

### 3.4. Application of Tomato Fiber in the Formulation of Tomato Sauce

**Figure 12:**
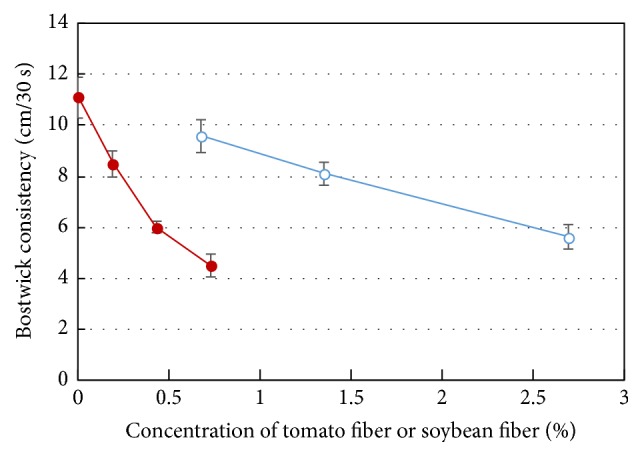
Bostwick consistency (cm/30 s) of tomato sauces prepared by using homogenized tomato fiber (0.19%–0.73%) or soybean fiber (0.67%–2.7%) using formula in Table 1. The red filled circles for tomato fiber; blue open circles for soybean fiber.

Dietary fibers such as soybean fiber are frequently added to produce tomato sauce. Thus, the effect of addition of homogenized tomato fiber or soybean fiber was measured and is presented in Figure 12. Bostwick consistency is employed in this section since it is more often used in the tomato industry than the rheological tests. A lower value of Bostwick consistency indicates a higher value of viscosity. As can be seen from this figure the addition of up to 0.5% (w/w) of tomato fiber could help the tomato sauce to achieve relatively high consistency. The amount of tomato fiber required would be one-third of the soybean fiber, to reach the same Bostwick consistency value. Typically, a tomato sauce with Bostwick consistency value of about 6–8 provides desirable texture or mouth feel of 0.2–0.5% dry fiber which is required.

A comparison of difference in color between the model tomato sauces prepared by using tomato fiber and soybean fiber is presented in Table 5. The Hunter color parameters (*L*, *a*, and *b*) and the ratio *a*/*b* are compared for these two formulations. A high value of *a*/*b* is desired in most tomato products. The *a*/*b* ratio containing tomato fiber is comparable but slightly higher compared to those containing soybean fiber. A slight decrease in total acidity was also observed in sauce samples containing tomato fiber.

## 4. Conclusions

The effects of high shear and high pressure homogenization on the morphological and rheological properties of tomato fiber were investigated. Both the high shear and high pressure homogenization processes made these suspensions much more homogeneous which enabled even distribution of fiber particles. Both the high shear and high pressure homogenization significantly (*p* < 0.05) increased the apparent viscosity of the tomato fiber suspensions. The apparent viscosity of the high pressure homogenized suspension was 10 times higher than that of unhomogenized one. The storage and loss modulus of the homogenized suspensions were higher than those of the unhomogenized one within the angular frequency range tested. The homogenized tomato fiber suspensions had more rigid structure compared to that of unhomogenized suspension and they resisted the deformation better (creep curve). The color and total acidity of model tomato sauce containing tomato fiber were more preferable than one containing soybean fiber at the same fiber content. The results presented in this paper indicate that tomato fiber can be potentially used as food ingredient such as thickener or stablizer.

## Figures and Tables

**Table 1:** Composition of tomato sauce prepared by using tomato paste and tomato fiber or soybean fiber.

Sample	Tomato paste	2.5% homogenized tomato fiber	Soybean fiber
P101	75 g	0	0
P102	76 g	8.33 g	0
P103	77 g	18.75 g	0
P104	78 g	32.14 g	0
P105	79 g	0	0.74 g
P106	80 g	0	1.48 g
P107	81 g	0	2.96 g

**Table 2:** The composition of tomato sauce prepared by using tomato fiber or soybean fiber, tomato paste, sugar, and salt.

Sample	Tomato paste (%)	2.5% tomato fiber homogenized (%)	Soybean fiber (%)	Sugar (%)	Salt (%)	Water (%)	Total
P110	80	--	2.5	6.2	0.9	10.4	100
P111	80	13	--	6.1	0.9	--	100
P112	75	16	--	8.1	0.9	--	100
P113	70	19	--	10.1	0.9	--	100

**Table 3:** Colorimetric parameters of unhomogenized tomato fiber suspensions at different concentration.

Conc.	*L*	*a*	*b*	*a*/*b*
0.25%	50.95 ± 1.79	4.63 ± 0.5	13.94 ± 0.27	0.33 ± 0.03
0.50%	40.35 ± 1.18	8.91 ± 0.25	14.80 ± 0.41	0.60 ± 0.033
1%	32.27 ± 0.02	11.08 ± 0.05	10.36 ± 0.01	1.07 ± 0.004
2%	32.23 ± 0.06	12.01 ± 0.02	10.39 ± 0.04	1.16 ± 0.004

**Table 4:** Power law tomato fiber suspension before and after mechanical treatment.

	Sample	*K* (Pa·s^n^)	*n*	*R* ^2^
Untreated	1%	0.73 ± 0.017	0.27 ± 0.0024	0.97
	0.5%	1.13 ± 0.031	0.12 ± 0.0013	0.82
	0.25%	0.01 ± 0.001	0.55 ± 0.0045	0.87
	0.1%	0.02 ± 0.001	0.36 ± 0.0028	0.85

High shear homogenized	1%	4.91 ± 0.038	0.07 ± 0.0006	0.85
	0.5%	2.65 ± 0.016	0.14 ± 0.0018	0.85
	0.25%	0.76 ± 0.008	0.18 ± 0.0014	0.75
	0.1%	0.18 ± 0.002	0.17 ± 0.0019	0.75

High pressure homogenized	1%	6.54 ± 0.045	0.04 ± 0.0004	0.75
	0.5%	8.82 ± 0.076	0.06 ± 0.0009	0.78
	0.25%	3.21 ± 0.032	0.04 ± 0.0006	0.74
	0.1%	0.43 ± 0.003	0.20 ± 0.0022	0.87

**Table 5:** The color parameters, pH, and total tomato sauce formulations prepared using high pressure homogenized tomato fiber or soybean fiber.

Sample	2.5% tomato fiber homogenized (%)	Soybean fiber (%)	*a*/*b*	*L*	*a*	*b*	pH	Total acid (%)
P110	--	2.5	2.17	24.38	30.05	13.82	4.09	1.9
P111	13	--	2.23	23.32	29.63	13.30	4.07	1.86
P112	16	--	2.27	23.61	29.12	12.84	4.07	1.79
P113	19	--	2.22	23.38	29.11	13.12	4.05	1.69
